# Real-time automatic temperature regulation during in vivo MRI-guided laser-induced thermotherapy (MR-LITT)

**DOI:** 10.1038/s41598-023-29818-z

**Published:** 2023-02-25

**Authors:** Manon Desclides, Valéry Ozenne, Pierre Bour, Thibaut Faller, Guillaume Machinet, Christophe Pierre, Stéphane Chemouny, Bruno Quesson

**Affiliations:** 1 CNRS, CRMSB, UMR 5536, IHU Liryc, University of Bordeaux, Bordeaux, France; 2 Certis Therapeutics, Pessac, France; 3 ALPhANOV, Talence, France

**Keywords:** Cancer, Surgical oncology, Biomedical engineering, Imaging techniques, Biological physics

## Abstract

Precise control of tissue temperature during Laser-Induced Thermotherapy (LITT) procedures has the potential to improve the clinical efficiency and safety of such minimally invasive therapies. We present a method to automatically regulate in vivo the temperature increase during LITT using real-time rapid volumetric Magnetic Resonance thermometry (8 slices acquired every second, with an in-plane resolution of 1.4 mmx1.4 mm and a slice thickness of 3 mm) using the proton-resonance frequency (PRF) shift technique. The laser output power is adjusted every second using a feedback control algorithm (proportional-integral-derivative controller) to force maximal tissue temperature in the targeted region to follow a predefined temperature–time profile. The root-mean-square of the difference between the target temperature and the measured temperature ranged between 0.5 °C and 1.4 °C, for temperature increases between + 5 °C to + 30 °C above body temperature and a long heating duration (up to 15 min), showing excellent accuracy and stability of the method. These results were obtained on a 1.5 T clinical MRI scanner, showing a potential immediate clinical application of such a temperature controller during MR-guided LITT.

## Introduction

Laser-Induced Thermotherapy (LITT) is a minimally invasive procedure that exploits localized heat deposition for several minutes to irreversibly alter pathological tissue. It is clinically applied for the treatment of glioblastoma or epilepsy in the brain^1,2^ and ablation of other types of cancer^3,4^ or diseases^5^. The procedure can be performed under real-time Magnetic Resonance (MR) thermometry monitoring to assess the thermal lesion boundaries by estimating the accumulated thermal dose from MR temperature maps computed with the Proton Resonance Frequency (PRF) shift method^6^. During the treatment, heat deposited by the device is dissipated from its source by tissue perfusion and thermal diffusivity. As a result, the laser energy is often set at a high value by the operator, which can result in tissue carbonization. In such a situation, tissue temperature can become hardly controllable due to modification of light absorption around charred tissues^7,8^. Moreover, in case of tissue desiccation or vaporization resulting from very high energy delivery, MR thermometry may become unreliable. Other therapeutic strategies rely on a moderate temperature increase of a few degrees above body temperature (around 40–43 °C) to stimulate the immune response for enhancing anti-tumor action^9,10^, to locally deliver chemotherapies encapsulated into thermosensitive nanocarriers^11,12^ or to spatially control activation of a gene of interest under a heat-sensitive promoter such as Hsp70^13^.

However, such LITT procedures require precise control of temperature distribution within the targeted tissue over time and in 3D. Indeed, the mere knowledge of the energy emitted by the device is insufficient because it poorly correlates with the actual temperature increase. The first issue is associated with precise probe positioning relative to the tumor, which is usually guided by intraoperative imaging. Nevertheless, light absorption by tissue together with micro- and macro-perfusion^14,15^ and thermal diffusivity remain unknown parameters that directly influence heat accumulation and dispersion in living tissues. These parameters are not only specific to each tissue but can also vary during treatment and be spatially heterogeneous within the pathological tissue and its environment.

A solution to overcome this problem is to automatically control the light energy delivered to the tissue during LITT using a closed-loop temperature algorithm based on real-time temperature sensor feedback^16,17^. This method offers the advantage of being fast and precise, although not being volumetric. Nevertheless, thanks to the recent development of electronic and computer equipment offering a markedly improved calculation speed, MRI thermometry methods have been developed to allow real-time volumetric temperature imaging of soft tissues on conventional clinical MRI systems with an update time of around one second even for moving organs^18,19^. Thereby, the design and development of automatic ablation processes can be envisioned by combining closed-loop methods with MR thermometry, allowing real-time and volumetric control of energy deposition during the treatment. This method can be associated with any thermal energy source (radiofrequency, focused ultrasound, microwave, laser) and was previously investigated for High-Intensity Focused Ultrasounds (HIFU)^20–24^. LITT has the advantage to be easily operated in an MRI environment as the only equipment inside the faraday cage is an optical fiber brought percutaneously into contact with the target region. There is no additional electromagnetic source of noise using this technique.

In the present study, we propose a method to automatically and dynamically adjust the delivered laser power to force temperature measured by real-time volumetric MR thermometry at the targeted region to follow a predefined temperature–time profile. The method is implemented on a clinical MRI scanner and evaluated on gel and a large animal model. First, a non-destructive initial shot is performed in a gel phantom to estimate the thermal parameters of the sample that are then used in the automatic temperature control algorithm. The target temperature–time profile consisted of three successive temperature increase plateaus of 100 s at 5 °C, 10 °C, and 15 °C. The same methodology is applied in a pig leg muscle in vivo, with an additional experiment consisting of a single temperature increase of 30 °C above body temperature for 700 s.

## Results

### Laser characterization

**Figure 1:**
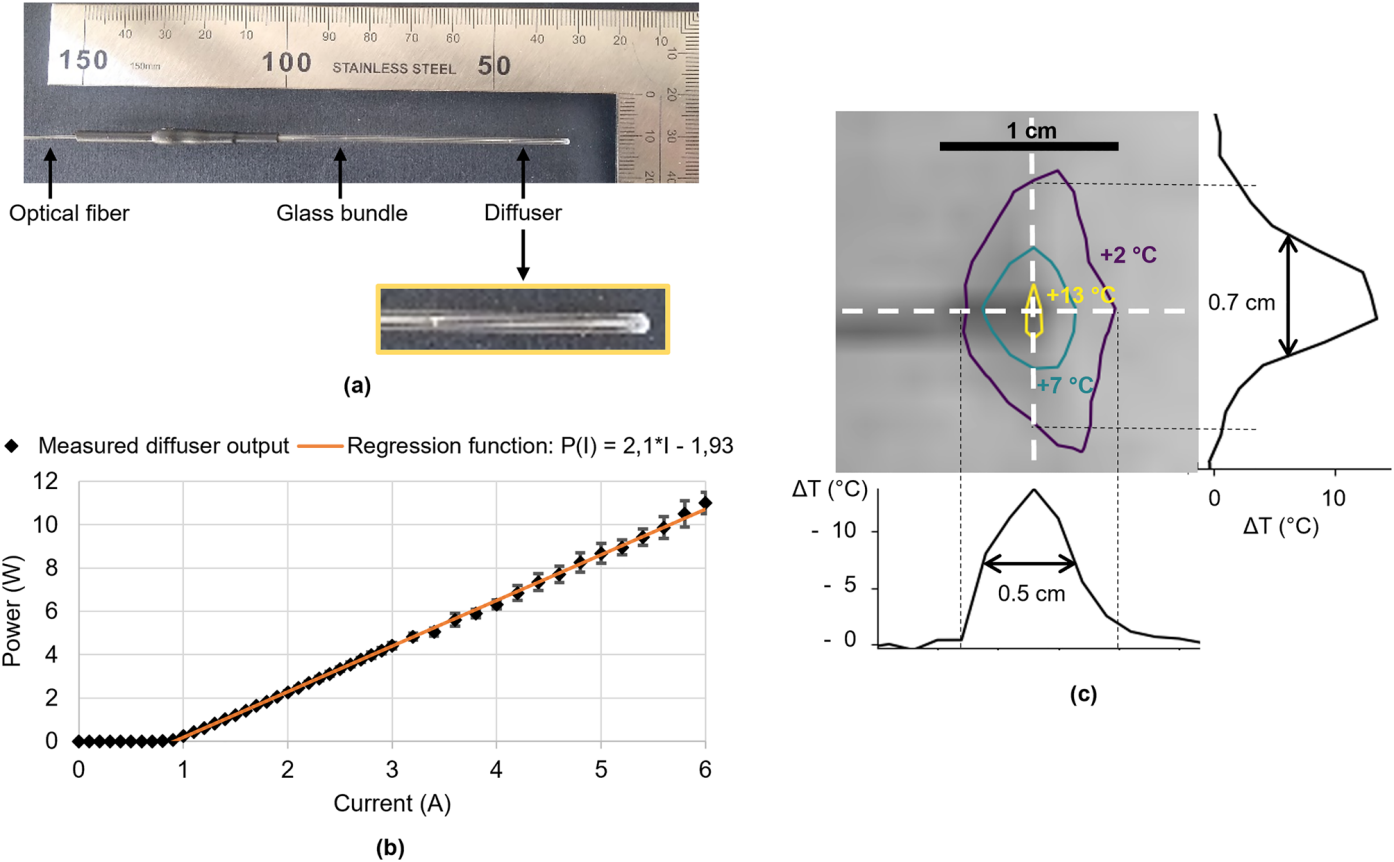
Laser device presentation. A laser probe photograph, with zoom-in on diffuser tip (**a**), its transfer function (Current (A) vs Power (W)) (**b**), and a spatial characterization of the energy deposit (**c**).

The laser device used in the study is composed of a laser diode source (976 nm wavelength, maximum output power of 27 W) connected to a 240 µm multi-mode optical fiber ended with a glass diffuser tip (1 cm long, 1.8 mm diameter) (see inset image in Fig. 1a). The curve displayed on the Fig. 1b shows the correspondence between input current (ranging 0 to 6 A) applied to the diode and the resulting output power measured with an integrating sphere photodiode power sensor. A linear fit was used to set the desired current in the experiments. Figure 1c displays an MR temperature map (isocurves of temperature increase) centered on the diffuser tip. Transverse and axial profiles are displayed lateral and below the figure with measured full width at half maximum.

### Gel phantom study

#### Thermodynamic parameters estimation

**Figure 2:**
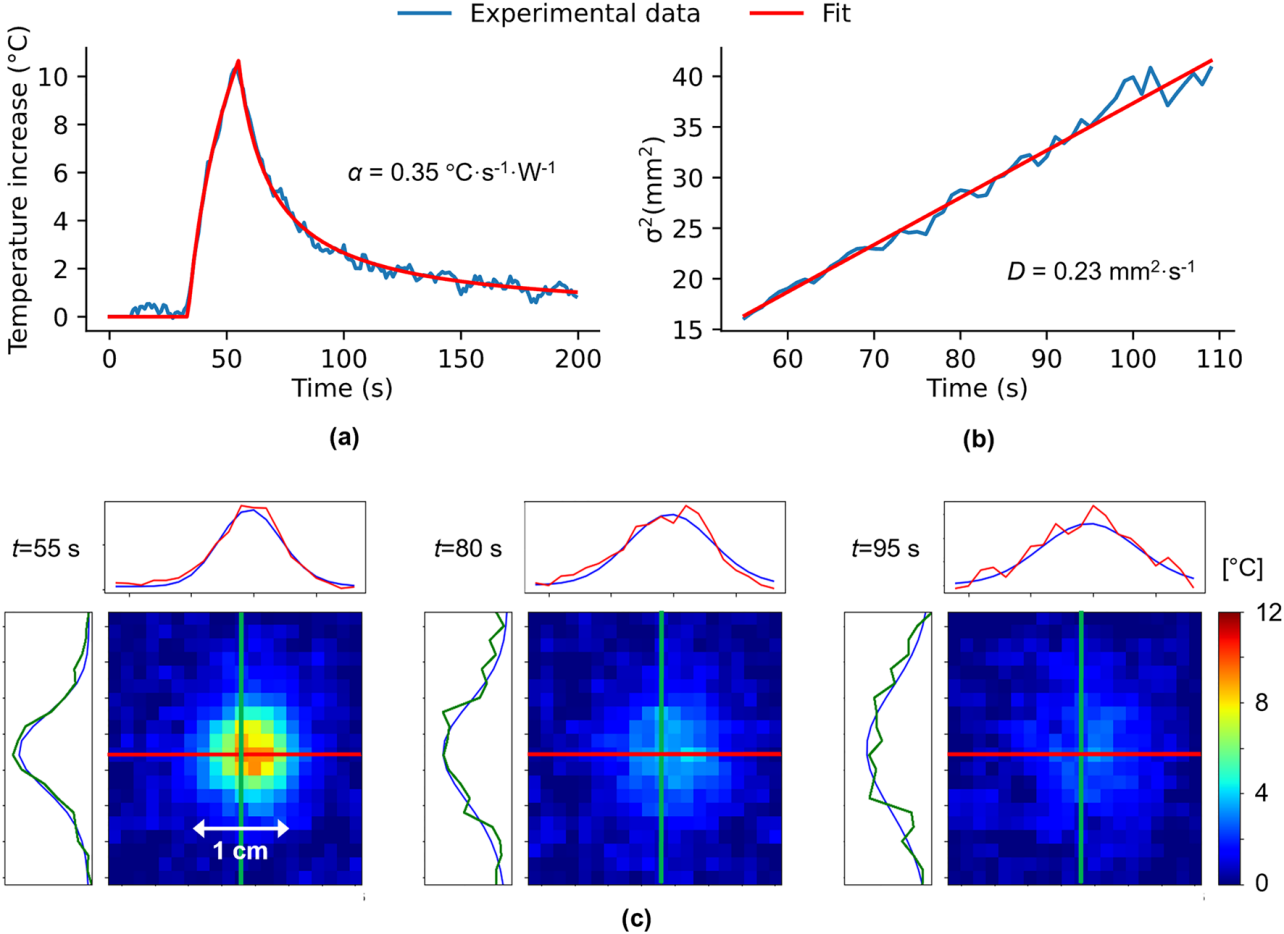
Estimation of absorption $\alpha$ (°C s^−1^ W^−1^) and diffusion $D$ (mm^2^ s^−1^) coefficients from the test shot in a gelatin gel. (**a**) The logarithmic fit of the focal light beam point temporal temperature profile to estimate $\alpha$. (**b**) The fit of the spatial heat Gaussian width temporal evolution to estimate $D$. (**c**) Temperature maps obtained at times $t$= [55, 80, 95] s and the resulting fit with a 2D Gaussian function (blue curves) compared to their spatial profile centered on the focal point (X direction in red, Y direction in green).

Figure 2 shows representative MR thermometry results of a short duration low intense laser emission (3 W during 20 s) into a gelatin phantom. The maximum temperature increase was less than 12 °C as seen in Fig. 2a, the blue curve representing the temperature evolution at the hottest point. The thermodynamic absorption coefficient $\alpha$ was estimated to $\alpha$ = 0.35 °C s^−1^ W^−1^ by fitting the experimental curve with Eq. (5) (red curve). The fitting routine converged in less than 6 s. The diffusion coefficient *D* was estimated by analyzing the temporal evolution of the temperature distribution during the cooling period (from $t$ = 55 s to $t$= 110 s). Three temperature maps recorded at different time points (55, 80, and 95 s) and resulting fit to a 2D Gaussian function are reported in Fig. 2c. Green curves (resp. red) are spatial temperature profiles corresponding to voxel values along green lines (resp. red) on images in the X (resp. Y) direction. Blue curves are the results of the fit corresponding to the position of green and red lines. The evolution of heating width is plotted as a function of time in Fig. 2b (blue curve) and was fitted with the linear relation resulting from Eq. (6) (red curve) to estimate the thermal diffusion coefficient $D$. In this example, we found $D$ = 0.23 mm^2^ s^−1^.

#### Automatic temperature regulation

**Figure 3:**
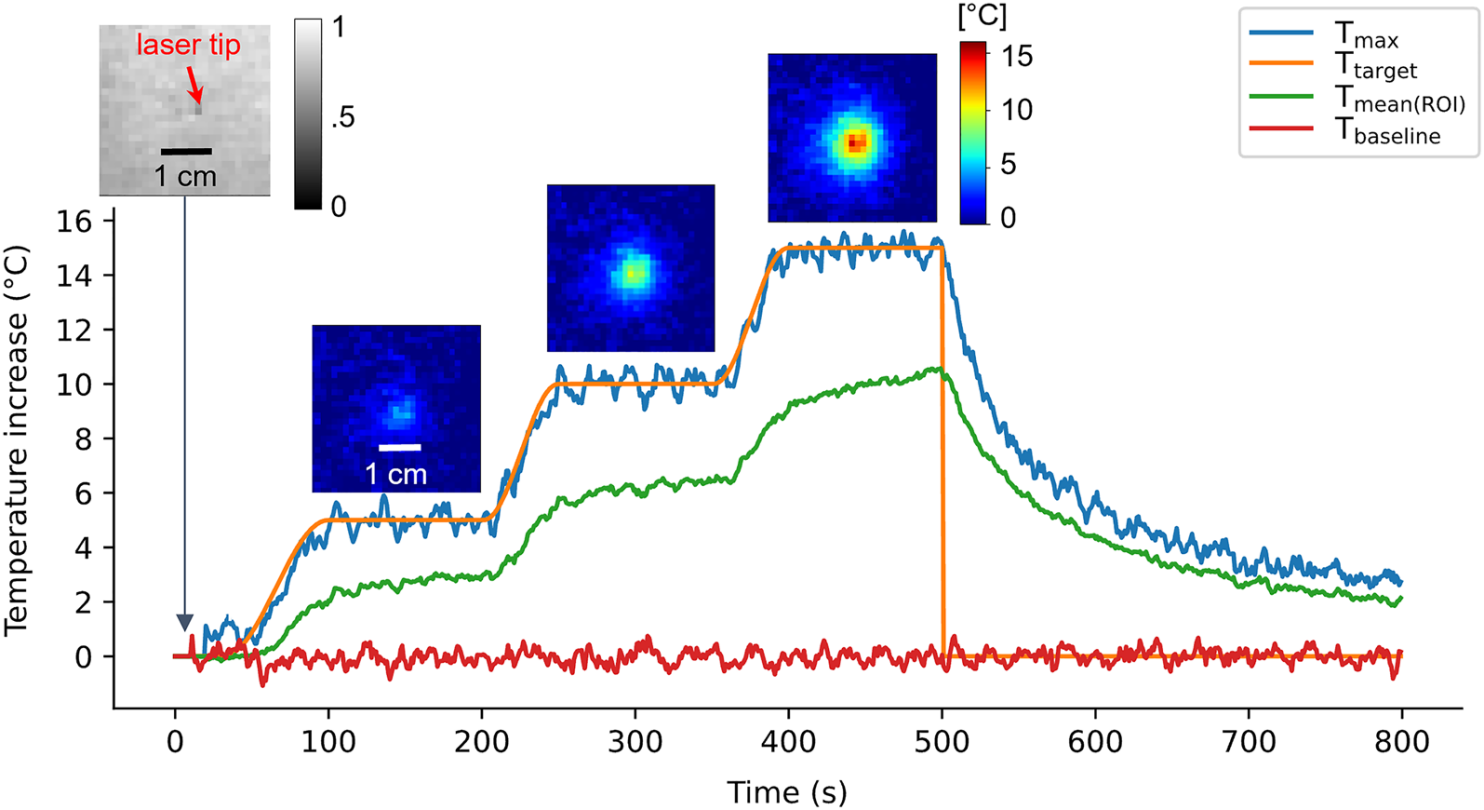
Example of an automatic temperature regulation during LITT in a gelatin gel, for a temperature–time profile showing 3 successive plateaus (5 °C, 10 °C, and 15 °C—100 s duration each). Target temperature profile (orange), maximal (blue), and mean (green) temperature curves over the ${3\times3\times3}$ voxels ROI are displayed. The red curve shows temperature evolution in a non-heated single voxel. Image insets: magnitude image (W&B left image) of the thermometry sequence and temperature maps obtained in the middle of each temperature plateau (3 colored right images, same color scale).

An example of automatic temperature regulation with our LITT device performed on a gelatin phantom with an initial temperature of 14 °C is presented in Fig. 3. Thermodynamic parameters used were those derived from the test shot heating presented in Fig. 2 and *q* was empirically chosen equal to 0.25 ${\text{s}}^{-1}$. The target temperature–time profile included 3 successive plateaus of 5 °C, 10 °C, and 15 °C, each lasting 100 s, respectively. Resulting regulated temperature over time using the temperature measured at the hottest point in the ${3\times3\times3}$ voxels regulation Region Of Interest (ROI) (blue curve) and its mean temperature (green curve) are displayed. We observe an excellent correspondence between target and maximal temperature values (Table 1, top part), with a mean difference and a Root Mean Squared Error (mean, RMSE) of (−0.06 °C, 0.45 °C) during the heating period and of (−0.01 °C, 0.33 °C), (0.03 °C, 0.39 °C) and (−0.05 °C, 0.31 °C) during each plateau.

**Table 1:** Summary of the regulation performance for in vitro and in vivo experiments. The mean differences between target and experimental temperature, together with RMSE at the control voxel are reported for each plateau of temperature increase and the total heating duration. The standard deviation of temperature (STD) in a non-heated voxel is also indicated to illustrate the stability of the thermometry method.

	Plateaus (°C)						Total heating duration
**In vitro experiments**	0 → 5	5	5 → 10	10	10 → 15	15	
Mean difference	–0.51	–0.01	–0.27	0.03	–0.27	–0.05	–0.06
RMSE	0.56	0.33	0.56	0.39	0.53	0.31	0.45
STD in a non-heated voxel	0.29	0.26	0.21	0.29	0.29	0.23	0.27
**In vivo experiments**	**0 → 5**	**5**	**5 → 10**	**10**	**10 → 15**	**15**	
Mean difference	−0.48	0	−0.17	−0.02	−0.19	0.01	−0.05
RMSE	0.74	0.66	0.79	0.77	0.92	1.02	0.82
STD in a non-heated voxel	0.44	0.44	0.36	0.46	0.45	0.52	0.47

	**0** → **30**	**30**					
Mean difference	−0.95	0.05					−0.1
RMSE	1.85	1.32					1.4
STD in a non-heated voxel	0.62	0.71					0.69

The red curve shows the temperature evolution of a non-heated single voxel to demonstrate the stability of temperature measurement over the time course (more than 12 min) of the experiment $({\sigma }_{baseline}$ = 0.27 °C). A magnitude image of the thermometry sequence before heating $(t$ = 12 s) and temperature maps obtained in the middle of each plateau ($t$ = [150, 300, 450] s) are presented in Fig. 3.

### In vivo study

#### Thermodynamic parameters estimation

**Figure 4:**
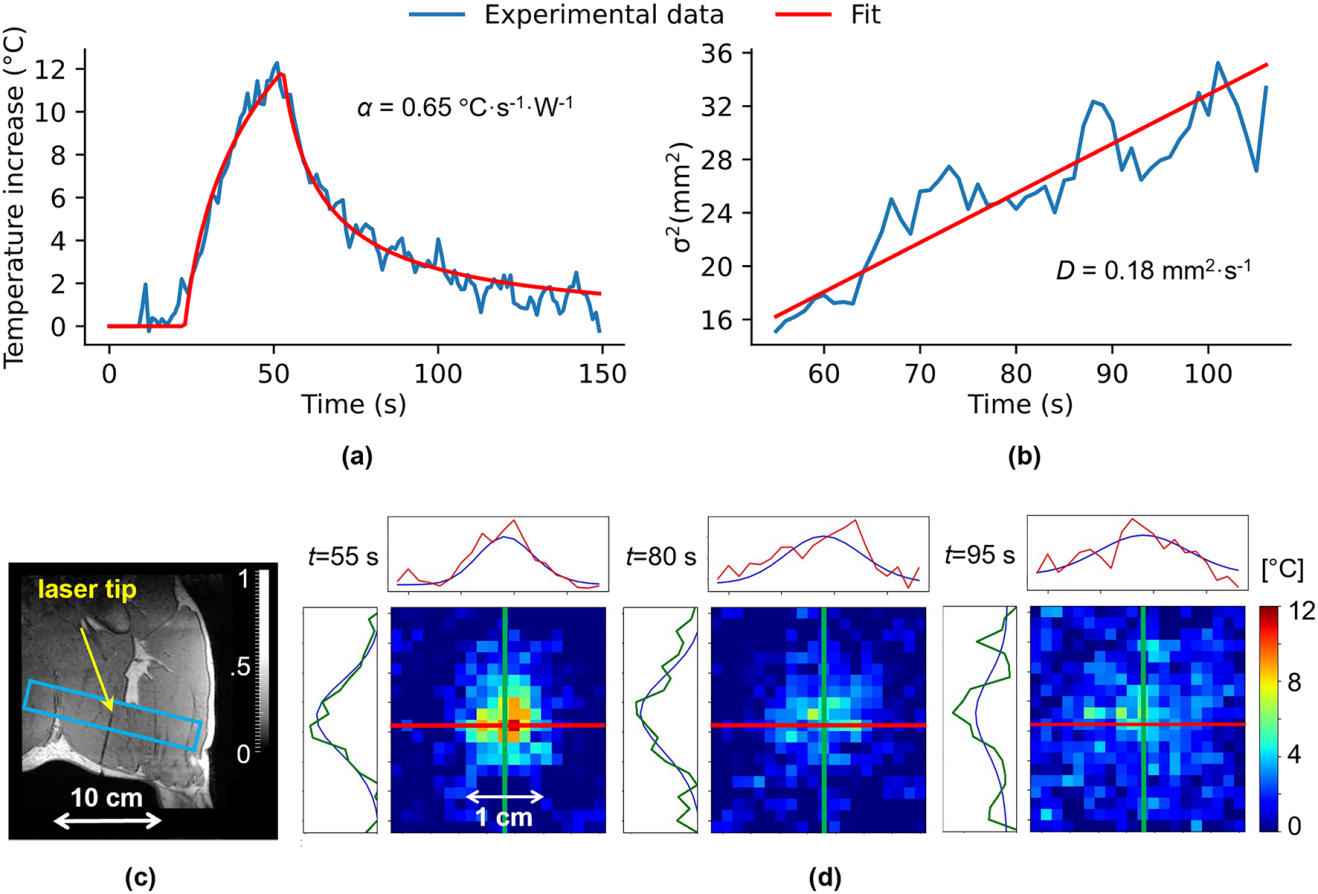
Estimation of absorption $\alpha$ ($^{\circ}{\text{C}}\,{\text{s}}^{-{1}}\,{\text{W}}^{-1}$) and diffusion $D$ (${\text{mm}}^{2}\,{\text{s}}^{-1}$) coefficients from the test shot in pig muscle. (**c**) Laser tip placement visualization (left leg) on a 3D MPRAGE coronal view with thermometry EPI stack of slices used in blue. (**a**) The logarithmic fit of the focal light beam point temporal temperature profile to estimate $\alpha$. (**b**) Temperature maps obtained at times $t$ = [55, 80, 95] s and the resulting fit with a 2D Gaussian function (blue curves) compared to their spatial profile centered on the focal point (X direction in red, Y direction in green). (**d**) The fit of the spatial heat Gaussian width temporal evolution to estimate $D$.

The automatic regulation algorithm was then evaluated in vivo in the leg muscle of an anesthetized pig. The laser probe position can be visualized in Fig. 4c (coronal view). The stack of slices for the thermometry sequence was positioned centered on the probe tip, represented as a blue rectangle in Fig. 4c. As for the experiment in the gelatin sample, $\alpha$ and $D$ parameters were estimated with an initial test shot (2 W during 30 s), leading to a 12 °C maximum temperature increase. Fit results for absorption and diffusion are shown in Fig. 4a and b, respectively. Thermodynamic parameters found for pig leg muscle were $\alpha =0.65$ $^{\circ}\text{C}\,{\text{s}}^{-1}\,{\text{W}}^{-1}$ and $D=0.18$ ${\text{mm}}^{2}\,{\text{s}}^{-1}$.

Supplementary figure Fig. S1 shows unfiltered and filtered temperature curves corresponding to the estimations of the thermodynamic parameters performed on the gel and the pig leg presented in Figs. 2 and 4 respectively. For both conditions, we observed a reduction of noise on temperature measurements and no temporal shift of the temperature curve.

#### Automatic temperature regulation

**Figure 5:**
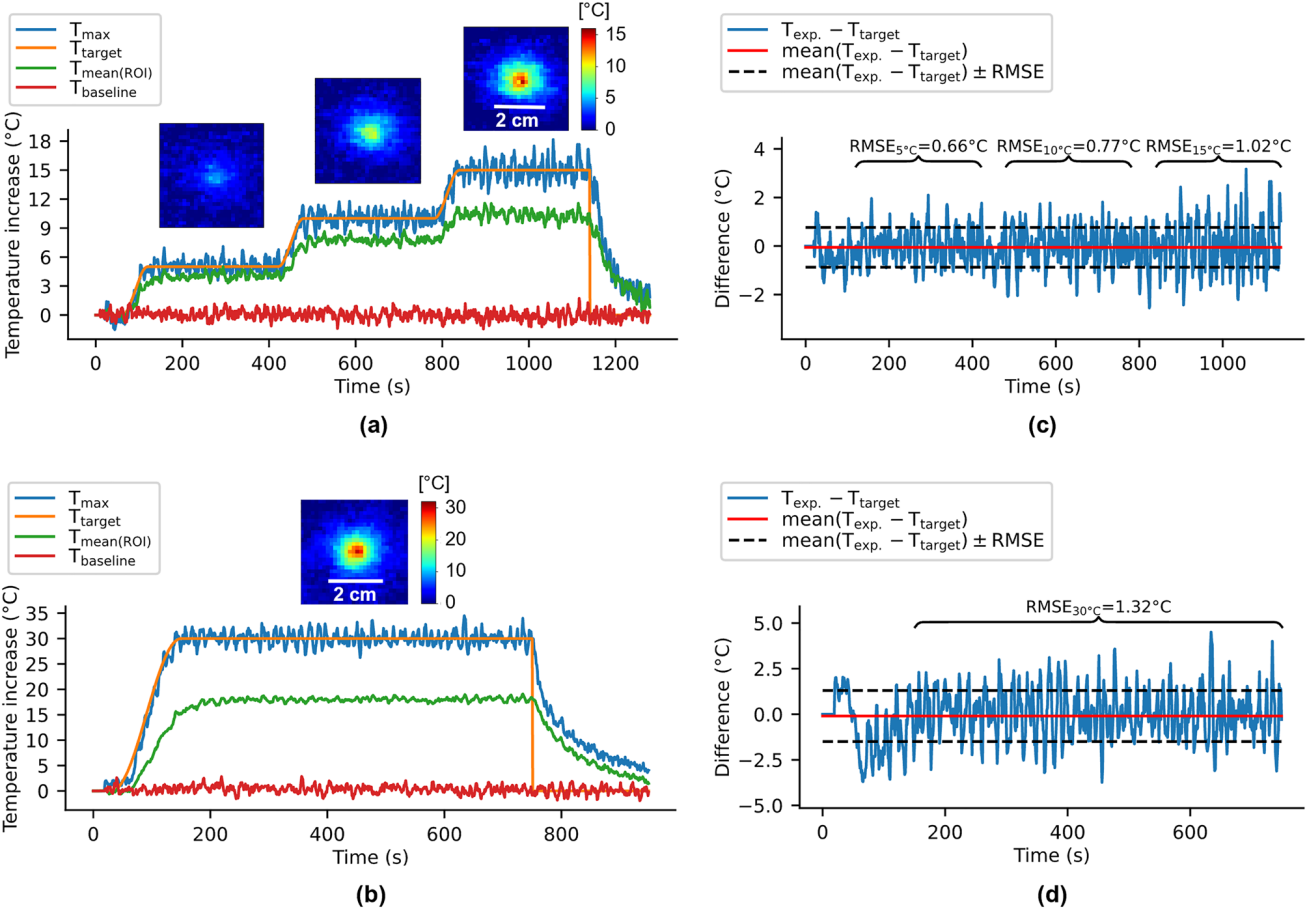
Examples of in vivo automatic temperature regulations during LITT. First column shows examples of automatic temperature regulations in a pig leg muscle, for 2 temperature–time profiles showing 3 successive plateaus (5 °C, 10 °C and 15 °C—300 s duration each) (**a**) and one unique plateau of 30 °C for 700 s (**b**). Target profile (orange), maximal (blue), and mean (green) temperature curves over the ${3\times3\times3}$ voxels ROI are displayed. The red curve shows temperature evolution in a non-heated single voxel. Image insets: temperature maps obtained in the middle of each temperature plateau (same color scale for each example). Graphs (**c**) and (**d**) in the second column show the difference between the experimental temperature and the target temperature, its mean, and its RMSE for example (**a**) and (**b**) respectively.

Two procedures performed on the pig muscle (${T}_{init}$= 38.6 °C) are presented in Fig. 5. The first experiment (a) had a temperature–time target profile showing 3 successive plateaus of 5 °C, 10 °C, and 15 °C each during 300 s (Fig. 5a, orange curve), and the second experiment (b) (performed at a different location) had a unique plateau of 30 °C for 700 s (Fig. 5b, orange curve). The maximal and the mean temperature in the ${3\times3\times3}$ voxels ROI are plotted in blue and green, respectively. We observe good stability of the regulation algorithm for both experiments (Table 1, bottom part). Red curves in both graphs show temperature evolution in a non-heated single voxel (${\sigma }_{baseline,a}$ = 0.47 °C, ${\sigma }_{baseline,b}$ = 0.69 °C). Images on Fig. 5 are temperature maps obtained in the middle of each plateau (${t}_{a}$= [300, 600, 1000] s and ${t}_{b}$ = 450 s). The difference between target and maximal temperature curves during the heating period are plotted on the right of Fig. 5, together with the mean (red lines) and RMSE values (black dot lines). Mean differences and RMSE (mean, RMSE) for the total heating duration of the experiment (a) are (−0.05 °C, 0.82 °C) and (0 °C, 0.66 °C), (−0.02 °C, 0.77 °C), and (0.01 °C, 1.02 °C), are values corresponding to each plateau. For experiment (b), values of mean difference and RMSE are (−0.1 °C, 1.4 °C) for the total heating duration and (0.05 °C, 1.32 °C) over the plateau of + 30 °C.

**Figure 6:**
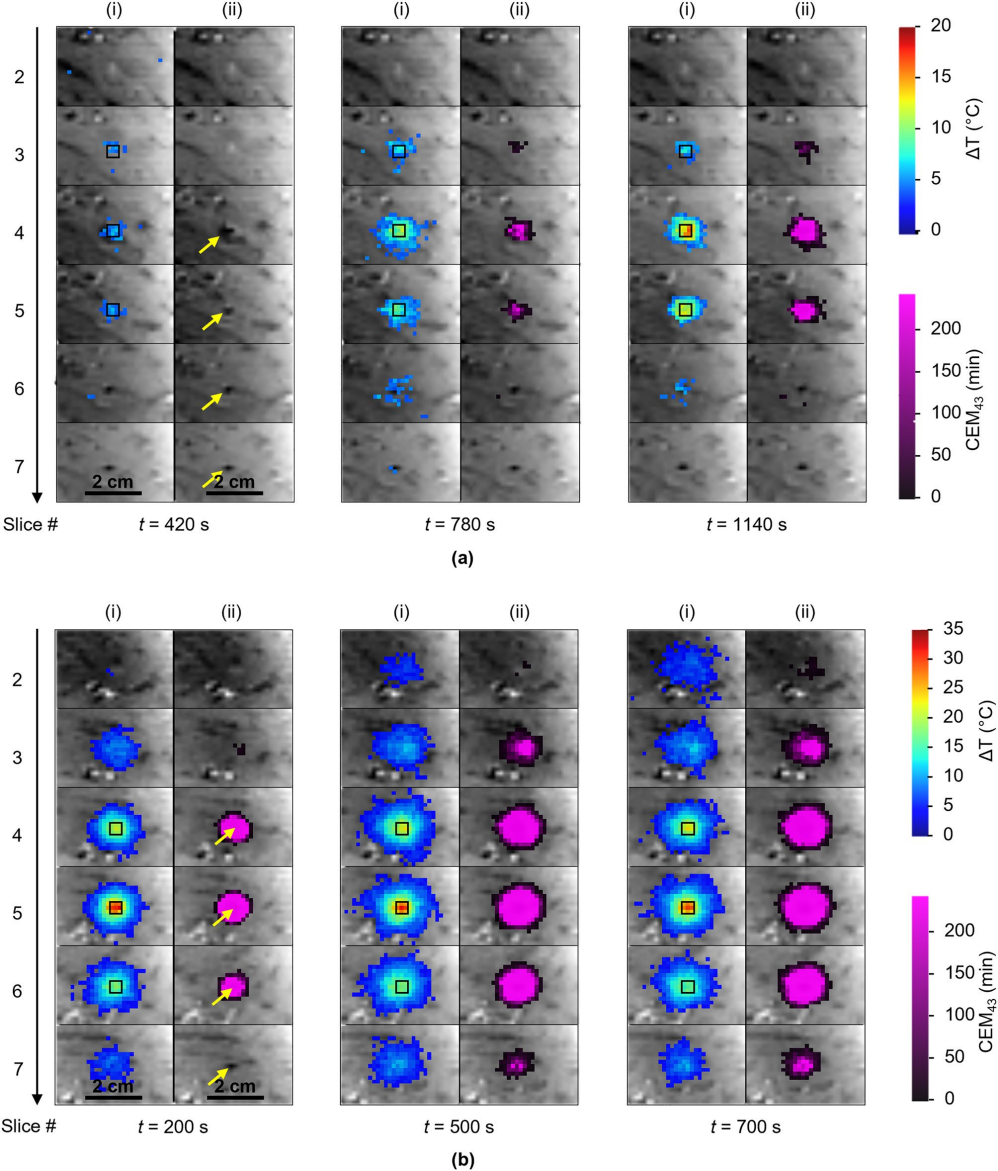
Temperature (i) and thermal dose (ii) images for the example of automatic temperature regulations during LITT in a pig leg muscle, for 2 temperature–time profiles showing 3 successive plateaus (5 °C, 10 °C, and 15 °C—300 s duration each) (**a**) and one unique plateau of 30 °C for 700 s (**b**) at different times ${t}_{a}$ = [420, 780, 1140] s et ${t}_{b}$ = [200, 500, 700] s, respectively. Yellow arrows show the laser probe on the magnitude images and black squares represent the ROI used for the automatic regulation.

Figure 6 shows the temperature and thermal dose images for six consecutive slices as a function of time, for experiments (a) and (b) presented in Fig. 5. This figure confirms that the maximal temperature increase remained inside the control ROI, since no hotspots were observed elsewhere. For experiment (a), only a limited number of voxels reached the lethal thermal dose in two consecutive slices, whereas for the experiment (b), the thermal dose progressively expanded in time over six slices, illustrative of the interest of temperature regulation for different thermotherapy strategies.

## Discussion

In this study, we demonstrate the feasibility of automatically regulating in real time the temperature during LITT ablation procedure. Issues associated with the absence of real-time temperature control have been highlighted by recent reviews on LITT advances^1,2,25–27^.

The proposed method was successfully evaluated in vitro in a gel and in vivo in the pig muscle. It was implemented on a clinical scanner (1.5 T) to demonstrate potential clinical application. It is based on a Proportional Integral Derivative (PID) controller integrating a physical model of temperature evolution as already proposed for HIFU^22^, combined with rapid and multi-slice MR thermometry^18^ (8 slices/s). Conventional low-pass filters usually introduce delays in the temperature evolution of the filtered data. To maintain the stability of the PID controller while ensuring accurate temperature input, a Kalman filter^28^ was included in the thermometry pipeline. The current implementation was shown to be efficient in reducing the temporal shift on filtered data and led to temperature uncertainty below 2 °C for both in vitro and in vivo experiments. It also had the advantage of adjusting its coefficients at each new acquired volume, allowing a noise reduction adapted to that contained in the current temperature signal. The implemented filter did not induce any lag (see Supplementary Figure S1). With such an implementation, a stable thermometry was achieved over long duration (15 min) procedures, which is a necessary condition for efficient monitoring or control of temperature distribution during a LITT procedure.

We showed that the control algorithm is working properly for different ranges of temperature increase, with values typically used for non-destructive procedures (a few degrees above body temperature) and for inducing thermal necrosis (+ 30 °C temperature increase). The proposed method would benefit a wide range of medical applications where precise control of tissue temperature rise is desirable^29,30^. Avoiding excessive temperature increase is also expected to reduce the risks of tissue carbonization that limits the spatial extent of the thermal lesion^31–33^.

To achieve such a temperature control, we first estimate the thermodynamic parameters $\alpha$ and $D$. A similar method was initially proposed for HIFU^34^ and was adapted and tested here for the laser source. For both in vitro and in vivo experiments, the maximal temperature reached 12 °C for only a few seconds, which is unlikely to create any thermal damage to the tissue. Under these experimental conditions, the fitting procedure worked properly and supplied input parameters to the PID controller. The diffusivity derived from the model was in the same range as those reported previously in ex vivo pig muscle^35^. In our model, perfusion was not taken into account although it could have been included in the model. However, this would require additional measurements which would increase the complexity of the calibration procedure. Moreover, it was demonstrated that perfusion acts as a scaling factor on the temperature amplitude but does not modify its spatial distribution^36^. Therefore, the perfusion term can be included in an apparent absorption coefficient that would represent the actual absorption attenuated by a term depending on perfusion. Using a simplified Bio-Heat Transfer Equation (BHTE) with only two parameters allows accounting for realistic tissue temperature change, with one term representing energy deposition ($\alpha$) and a second term accounting for the spatial spread of temperature ($D$). Taking into consideration the update rate of MR thermometry, we considered this simplified approach of practical interest. For both in vitro and in vivo experiments, we show that the experimental temperature follows the target profile at the hottest voxel.

In our model, we hypothesize that diffusion is isotropic over the target region. This assumption may not be valid in targets located at the border between tissues having different heat conductivities. The calculation of a diffusion map on the target region and its surroundings may be necessary to provide a more precise knowledge of the temperature distribution if a control at other places than at the hottest point is desired. Moreover, absorption and diffusion coefficients were assumed to remain constant during the procedure, which is unlikely to occur when coagulation necrosis is induced^37^. However, the PID controller was shown robust to precisely control the temperature even in an ablative regime such as in experiment (b) (Fig. 5d) where the thermal dose reached the lethal threshold (Fig. 6b, ii).

Although the Kalman filter is efficient and fast, we can still notify that the dependence of the filter on the temperature derivative can create delays in case of abrupt changes in the slopes of the temperature curves. For these reasons, we defined target temperature profiles with maximal increase rates of 15 °C/min, followed by plateaus of several minutes. Under these experimental conditions, we demonstrate that precise temperature control can be achieved even at a + 30 °C temperature increase (68 °C absolute temperature) in the muscle. Considering that current clinical LITT treatments last several minutes, controlling the maximal heating rate has a negligible impact on the overall procedure duration. However, the Kalman filter may be refined by adding a power term indication (its value or an on/off state flag) in the state transition model to allow the filter prediction to consider a potential abrupt change in the temperature slope and thus reduce the derivative propagation effect. In our experimental conditions, such optimization was not considered necessary given the quality of temperature regulation.

A non-negligible point to address is the problem of potentially important MRI signal amplitude reduction at very high tissue temperatures, due to T2* shortening and T1 lengthening. Such a reduction increases the uncertainty of the MRI phase estimate and consequently the temperature standard deviation.

In Fig. 5c, d, we observed that the RMSE values measured on plateaus increase with the target temperature from 0.66 °C (5 °C plateau) to 0.77 °C (10 °C plateau) and 1.02 °C (15 °C plateau). For a target temperature of 30 °C, the RMSE reached 1.32 °C, which is approximately twice the standard deviation of the temperature baseline (0.73 °C). However, this RMSE value was considered acceptable for an increase of tissue temperature up to 30 °C and did not alter the quality of temperature regulation, as shown by the mean error remaining below 0.1 °C.

Our algorithm excludes voxels under a predefined Signal to Noise Ratio (SNR) threshold (here 5% of the maximum). Reduction of the SNR during the procedure may thus lead to a shift to a different voxel in the ROI used for control. The back-controlled point then no longer corresponds to the hottest point located in the immediate vicinity of the energy source. This lack of information may be compensated either by filling the missing voxels with real-time simulated temperature or by adapting the power to the position of the chosen point regarding the heat source geometry. Alternatively, the predefined threshold could be dynamically adjusted.

This study shows potential great benefits for improving clinical LITT. A combination of rapid and volumetric thermometry associated with automatic regulation of tissue temperature is expected to avoid excessive temperature increase that is often associated with interruption of the treatment in up to about 30% of the cases for LITT ablation in the brain^38^. The 3D coverage of our MR thermometry pipeline outperforms current state-of-the-art MR-based temperature imaging techniques used in the clinic^39^, where only a limited number of slices (usually 3 orthogonal slices) are centered on the device, making precise visualization of the 3D heating pattern impossible. Under the tested conditions (in the leg muscle of an anesthetized pig), no motion is expected to occur, and reliable thermometry was reported over 15 min duration, which is long enough to monitor conventional clinical LITT ablation procedures. However, in the case of subject motion during the acquisition, artifacts on thermometry may occur and thus alter the quality of the regulation algorithm which results in an important energy delivery by the generator. A similar real-time thermometry pipeline was previously evaluated on the heart during catheter-based radiofrequency ablation and showed a temperature standard deviation below 2 °C on most of the pixels covering the myocardium^40^. Additional control steps can also be included in the real-time pipeline to limit the maximal allowed delivered power to reduce the risks of creating susceptibility artifacts.

## Methods

### Gel preparation

A gelatin gel phantom (3.0%) was made by adding 24 g of gelatin powder (Sigma-Aldrich, U.S.A.) in 800 mL of distilled water. The solution was heated in a microwave oven at 800 W stirring occasionally until ebullition (approximately 5 min), before cooling down to room temperature.

### Animal experiments

In vivo experiment was performed in the leg muscle of a pig (n = 1). The protocol was approved by the ethics committee Comité d’Ethique en Expérimentation Animale of Bordeaux n°50 (CEEA50, France) and was performed in accordance with European rules for animal experimentation. All authors complied with the ARRIVES guidelines. The animal was sedated by intramuscular injection of Ketamine (10–20 mg/kg), acepromazine (0.1 mg/kg), and Buprénorphine (9 μg/kg) and anesthetized by an intravenous injection of Propofol (1–2 mg/kg). The animal was intubated after induction of anesthesia, positioned supine in the MRI scanner, and ventilated using an MR-compatible ventilator (Aestivia, General Electric, Fairfield, CT, U.S.A.) at 15 breaths per minute. Anesthesia was maintained during the whole experiment by continuous breathing of isofluorane (1.5–3%) in a mixture of air/oxygen 50/50. Cardiac rhythm and intra-arterial pressure were monitored during the entire experiment (Carescape, General Electric, Fairfield, CT, U.S.A.).

### Laser device

The prototype laser device (ALPhANOV, France) consists of a fully integrated laser diode (976 nm, maximum average power of 27 W) module interfaced with an electronic board. The optical fiber connected to the laser unit was a multimode fiber of 240 µm core diameter and a numerical aperture of 0.22. The distal part of the device consisted of a 1 cm long homemade diffuser inserted in a glass bundle (1.8 mm diameter) to protect the fiber. Measurement of the laser output power was done using an integrating sphere photodiode power sensor (In-Ga-As, 900 to 1650 nm, 20 W, Thorlabs, U.S.A.) coupled with a digital handheld optical power and energy meter console (Thorlabs, U.S.A.). A calibration curve (output power versus applied current) was obtained by measuring output power for different input currents (three measurements for each condition, averaged together). The curve was then fitted by a linear function. The spatial distribution of the heating produced by the laser was obtained by heating a gel phantom at 3 W for 30 s under MR thermometry. During experiments, the probe tip was inserted into the samples with help of a holding catheter to guide the introduction. The laser unit was controlled via a USB serial connection from the Gadgetron^41^ framework used for online MR-image reconstruction and processing of temperature images. It allows setting the desired laser output power by adjusting the current applied to the photodiode, together with the onset time and duration of laser emission.

### MR-imaging protocol

MRI data were collected on a 1.5 T clinical MRI (Avanto, Siemens Healthineers). First, scout images were acquired on the subject to approximately locate the laser probe. Then, a 3D MPRAGE sequence was run with the following acquisition parameters (TI = 1000 ms, TE = 3 ms, TR = 2000 ms, FA = 15°, Field Of View (FOV) = 192 mm × 162 mm × 240 mm, 1 mm isotropic voxel size). The 3D volume was analyzed on the MRI console and images were reformatted to visualize the laser tip trajectory in two perpendicular projections. MR temperature images were obtained using a multi-slice single-shot echo planar imaging (EPI) sequence^18,42^ positioned perpendicular to the laser probe from the two selected orthogonal slices from the 3D MPRAGE sequence. A stack of 8 slices was acquired every second using the following parameters: TE = 21 ms, TR = 1000 ms, FA = 70°, 1.4 mm × 1.4 mm × 3 mm voxel resolution, GRAPPA acceleration = 2, partial Fourier = 6/8, bandwidth/pixel = 1445 Hz. The FOV was 180 mm × 180 mm for phantom studies and 160 mm × 160 mm for in vivo experiments. Two saturation slabs of 50 mm each were positioned on each side of the FOV in the phase encoding direction to avoid folding artifacts. MR-signal was acquired using a 4-channel array coil and two elements of the spine coil surrounding the subject. In total, 12 channels were used for image reconstruction. Raw data were streamed to a real-time reconstruction and thermometry pipeline^41^. The reference phase image used for the PRF-based (using a PRF constant of −0.0094 ppm/°C) thermometry reconstruction was created by averaging the 10 first dynamic acquisitions for each slice. A phase drift correction based on a spatio-temporal first-order polynomial function was applied on images with a high SNR (threshold set to 5% of the maximum of the average magnitude over 20 consecutive frames) and temperature comprised between two predefined thresholds (${T}_{min}<T<{T}_{max}$) initialized to -5 °C and + 5 °C, respectively. The estimation was done on each slice with a 20 dynamics temporal window and correction coefficients were continuously updated.

A temporal Kalman filter^28^ was applied to reduce noise on temperature estimates, by considering the temperature in each voxel at time $t+1$ as a function of its temperature $T\left(t\right)$ and its temporal derivative $\dot{T}\left(t\right)$ at time $t$, as described in the following transfer Eq. (1):

1 $$\left(\genfrac{}{}{0pt}{}{T\left(t+1\right)}{\dot{T}\left(t+1\right)}\right)=F\cdot \left(\genfrac{}{}{0pt}{}{T\left(t\right)}{\dot{T}\left(t\right)}\right)$$

with $$F=\left(\begin{array}{cc}1&\Delta t\\ 0& 1\end{array}\right)$$ the state-transition model matrix, and $\Delta t$ the temperature time resolution. Process noise covariance used for the implementation of this Kalman filter was set constant to ${\sigma }_{process}^{2}$ = 0.001 °C whereas the measurement noise covariance ${\sigma }_{measurement}^{2}$ was adapted to each acquisition by computing for each voxel the variance of the temperature temporal evolution during the 20-first temperature dynamic acquisitions.

Temperature and thermal dose images (with a Cumulative Equivalent Minutes at 43 °C (${\text{CEM}}_{43}$) threshold taken at 240 min) were displayed in real-time (Certis Solution, Certis Therapeutics, France).

### Automatic temperature regulation algorithm

Dedicated processing was implemented in the thermometry pipeline to compute and update the laser power on the fly according to MR temperature measurements. The controller is a proportional-integral-derivative (PID) algorithm based on Eq. (2), with $\xi \left(\overrightarrow{r},t\right)$ the difference between the target temperature ${T}_{t}\left(\overrightarrow{r},t\right)$ and the measured one ${T}_{m}\left(\overrightarrow{r},t\right)$ at a time $t$ and a location $\overrightarrow{r}$ and $-q/2$ the unique root of this second order polynomial function, ensuring stable convergence of the difference $\xi \left(\overrightarrow{r},t\right)$ toward 0 (i.e. ${T}_{m}\left(\overrightarrow{r},t\right)= {T}_{t}\left(\overrightarrow{r},t\right)$):

2 $$q\xi \left(\overrightarrow{r},t\right)+\frac{{q}^{2}}{4}{\int }_{0}^{\tau }\xi \left(\overrightarrow{r},t\right)d\tau +\frac{\partial \xi \left(\overrightarrow{r},t\right)}{\partial t}=0.$$

The presence of those three terms (proportional, integral, and derivative) allows the controller to take into account the current error, the sum of past errors, and the evolution of temperature at the next measurement time, respectively. The parameter $q$ was tuned to adapt the response time of the controller ${t}_{r}=2/q$ so that ${t}_{r}$ is several times greater than the latency time of the system comprising both measurement and computational times. In the experiments presented here, $q$ was set to 0.25 ${\text{s}}^{-1}$ since the update time of the thermometry was 1 s.

To calculate the power necessary for the temperature regulation, we used the BHTE^43^ (Eq. 3), which links the power applied $P\left(\overrightarrow{r},t\right)$ at a time t and at a location $\overrightarrow{r}$ on a tissue with thermodynamics absorption $\alpha$ ($^{\circ}{\text{C}}\,{\text{s}}^{-1}\,{\text{W}}^{-1}$) and diffusion $D$ (${\text{mm}}^{2}\,{\text{s}}^{-1}$) coefficients to its spatial and temporal heat variations ${\nabla }^{2}{T}_{m}\left(\overrightarrow{r},t\right)$ and $\partial {T}_{m}\left(\overrightarrow{r},t\right)/\partial t$ respectively:

3 $$\frac{\partial {T}_{m}\left(\overrightarrow{r},t\right)}{\partial t}=D\cdot {\nabla }^{2}{T}_{m}\left(\overrightarrow{r},t\right)+\alpha \cdot P\left(\overrightarrow{r},t\right).$$

In this implementation, perfusion was intentionally not included to avoid complex implementation and estimation of its value. Combining Eqs. (2) and (3) leads to the following expression (Eq. 4) allowing computation of the output power $P$ to be applied from time $t$ to time $t+\Delta t$ to force the temperature ${T}_{m}\left(0,t+\Delta t\right)$ to follow the target temperature ${T}_{t}\left(0,t+\Delta t\right)$ at position $\overrightarrow{r}=\overrightarrow{0}$:

4 $$P\left(t\to t+\Delta t\right)=\frac{1}{\alpha }\left[\frac{\partial {T}_{t}\left(t\right)}{\partial t}-D\cdot {\nabla }^{2}{T}_{m}\left(t\right)+q\left[{T}_{t}\left(t\right)-{T}_{m}\left(t\right)\right]+\frac{{q}^{2}}{4}{\int }_{0}^{\tau }\left[{T}_{t}\left(t\right)-{T}_{m}\left(t\right)\right]d\tau \right].$$

The voxel used for automatic regulation $T\left(\overrightarrow{r}=\overrightarrow{0}\right)$ was dynamically updated considering that the maximum value voxel of current temperature maps corresponds to the current source point. To avoid noisy voxel selection as the source point, we defined before the ablation procedure a ${3\times3\times3}$ voxels ROI centered on the maximum heated voxel using an initial low power shot (See Estimation of Thermal Parameters in the next part). The ROI was dynamically refreshed to remove voxels with low SNR (SNR < 5% of the maximum of the average magnitude over 20 consecutive frames) and with a temperature standard deviation superior to 1 °C, a signal loss being a consequence of tissue dehydration and therefore thermometry precision reduction potentially leading to instability of the regulation algorithm. Terms of Eq. (4) were directly calculated numerically from temperatures maps and the Laplacian term ${\nabla }^{2}{T}_{m}\left(0,t\right)$ was computed with the finite elements method over the updated ROI for each frame and temporally averaged from times $t$ to $t-4$ with the following weights: w = [1, 4, 6, 4, 1], using the ${\left(t-2\right)}$th resulting Laplacian value for the ${t}$th power computation. The regulation algorithm was implemented in a dedicated gadget written in C++ and incorporated into the existing thermometry pipeline developed under the Gadgetron^41^ framework. All experiments were performed with an Intel Xeon® W-2275 CPU @ 3.30 GHz × 28 cores.

### Estimation of thermal parameters

Before proceeding with the automatic temperature regulation, an initial shot was performed to locate the heated area and estimate the thermodynamic parameters. For this purpose, a continuous wave low-power laser emission was applied for a few seconds under MR thermometry. The voxel showing the maximal temperature value was determined and the ${3\times3\times3}$ ROI used for temperature regulation (as explained in the Automatic Temperature Regulation Algorithm part above) was automatically centered on this voxel. Determination of the absorption coefficient $\alpha$ resulted from a fit of temperature evolution at the hottest point^34^ with Eq. (5):

5 $$T\left(t\right)=\left\{\begin{array}{cc}0&\quad {if} t\le {t}_{0}\\ \alpha \cdot P\cdot \tau \cdot ln\frac{t-{t}_{0}+\tau }{\tau } &\quad {if} {t}_{0}<t\le {t}_{1}\\ \alpha \cdot P\cdot \tau \cdot ln\frac{t-{t}_{0}+\tau }{t-{t}_{1}+\tau }&\quad {if} t\ge {t}_{1}\end{array}\right.$$

where ${t}_{0}$ and ${t}_{1}$ are the start and stop times (s) of laser emission, $P$ (W) the laser power, and $\tau$ (s) the characteristic diffusion time of the system.

During the cooling phase, only the diffusion term remains in Eq. (3). It has been demonstrated^34^ that by applying a Fourier Transform on the spatial dimensions of Eq. (3) one can obtain a first-order differential equation whose inverse Fourier transform of its solution is a Gaussian function given in Eq. (6):

6 $$T\left(\overrightarrow{r},t\right)={T}_{0}\frac{1}{\sigma(t) \sqrt{2\pi }}{e}^{-\frac{{r}^{2}}{2{\sigma(t) }^{2}}},$$

were ${\sigma }^{2}\left(t\right)=2Dt$ varies linearly in time with a slope equal to $2D$. Thus, a fit of the temperature maps was done during the cooling period with a 2D Gaussian function (with ${\sigma }_{x}={\sigma }_{y}=\sigma$ assuming a constant value of $D$ over the heated area) on the hottest slice for each dynamic during the cooling period, and the resulting standard deviation values were plotted as a function of time. The curve was then fitted to a linear function to estimate $D$. Data were processed in homemade code written in Python language. $\alpha$ and $D$ were then set as input parameters into the regulation algorithm in Eq. (4).

### Analysis of the efficiency of temperature regulation

To evaluate the efficiency of the regulation algorithm, the difference between the target and experimental temperature curves was computed as a function of time during the heating period (i.e., target temperature $\ne$ 0). Mean difference value and RMSE were calculated for the resulting curve and each temperature plateau, to investigate whether the efficiency varies depending on the value of the target temperature. The standard deviation of baseline temperature measurements was also calculated to compare the RMSE of the controller and the measured temperature uncertainty. Data were processed using dedicated Python scripts.

## Supplementary Information

Supplementary Information.

## Data Availability

The datasets generated and analyzed during the current study are available in the Zenodo repository https://doi.org/10.5281/zenodo.7334405.
