# Supplementary material for: Real-time automatic temperature regulation during in vivo MRI-guided laser-induced thermotherapy (MR-LITT)
Source: Sci Rep. 2023 Feb 25;13:3279. doi: 10.1038/s41598-023-29818-z (PMC9968334; doi:10.1038/s41598-023-29818-z)
Supplement: Supplementary file 1 — Supplementary Information. [file 41598_2023_29818_MOESM1_ESM.pdf]

Real-time Automatic Temperature Regulation During In Vivo MRI-guided Laser Induced Thermotherapy (MR-LITT)

Manon Desclides[1,2,*], Valéry Ozenne[1], Pierre Bour[2], Thibaut Faller[2], Guillaume Machinet[3], Christophe Pierre[3], Stéphane Chemouny[2], Bruno Quesson[1]

[1]University of Bordeaux, CNRS, CRMSB, UMR 5536, IHU Liryc, Bordeaux, France
[2]Certis Therapeutics, Pessac, France
[3]ALPhANOV, Talence, France
[*]manon.desclides@u-bordeaux.fr

Supplementary Figures:

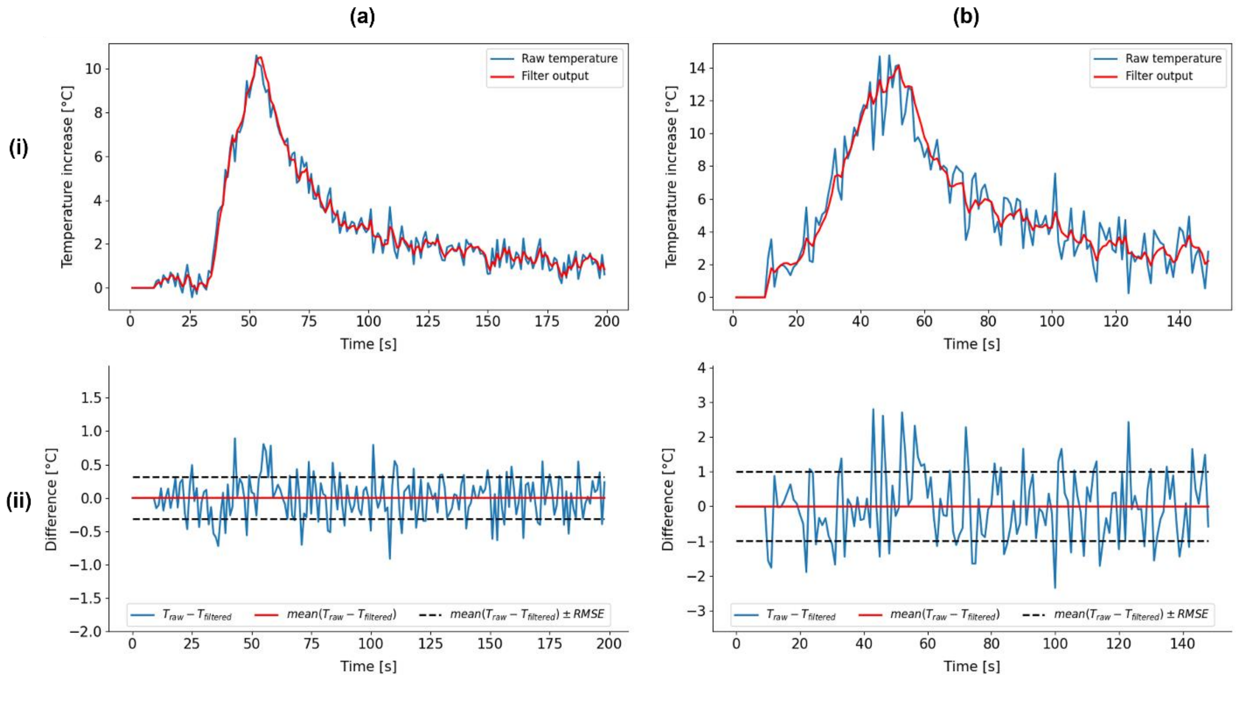

Supplementary figure S1: Examples of thermometry Kalman filtering. (i) shows Kalman filtered thermometry data (red curves) of the gelatin sample (a) and the pig muscle sample (b) raw temperature measurement (blue curves). (ii) shows the difference between the raw temperature and the filtered temperature, its mean and its RMSE for each example
